# A Mitochondrial Localized Chaperone Regulator OsBAG6 Functions in Saline-Alkaline Stress Tolerance in Rice

**DOI:** 10.1186/s12284-024-00686-z

**Published:** 2024-01-22

**Authors:** Jie Wang, Min Ao, Ao Ma, Jinlei Yu, Peng Guo, Shuangzhan Huang, Xiaoyuan Peng, Dae-Jin Yun, Zheng-Yi Xu

**Affiliations:** 1 Key Laboratory of Molecular Epigenetics of the Ministry of Education (MOE), Northeast Normal University, Changchun, 130024 China; 2 Jilin Province Engineering Laboratory of Plant Genetic Improvement, College of Plant Science, Jilin University, Changchun, 130062 China; 3 Department of Biomedical Science and Engineering, Konkuk University, Seoul, 132‐798 South Korea

**Keywords:** Rice, Chaperone regulator, Saline-alkaline stress, Plant height, Grain size

## Abstract

B-cell lymphoma 2 (Bcl-2)-associated athanogene (BAG) family genes play prominent roles in regulating plant growth, development, and stress response. Although the molecular mechanism underlying BAG’s response to abiotic stress has been studied in *Arabidopsis*, the function of OsBAG underlying saline-alkaline stress tolerance in rice remains unclear. In this study, OsBAG6, a chaperone regulator localized to mitochondria, was identified as a novel negative regulator of saline-alkaline stress tolerance in rice. The expression level of *OsBAG6* was induced by high concentration of salt, high pH, heat and abscisic acid treatments. Overexpression of *OsBAG6* in rice resulted in significantly reduced plant heights, grain size, grain weight, as well as higher sensitivity to saline-alkaline stress. By contrast, the *osbag6* loss-of-function mutants exhibited decreased sensitivity to saline-alkaline stress. The transcriptomic analysis uncovered differentially expressed genes related to the function of “response to oxidative stress”, “defense response”, and “secondary metabolite biosynthetic process” in the shoots and roots of *OsBAG6-*overexpressing transgenic lines. Furthermore, cytoplasmic levels of Ca^2+^ increase rapidly in plants exposed to saline-alkaline stress. OsBAG6 bound to calcium sensor OsCaM1-1 under normal conditions, which was identified by comparative interactomics, but not in the presence of elevated Ca^2+^. Released OsCaM1-1 saturated with Ca^2+^ is then able to regulate downstream stress-responsive genes as part of the response to saline-alkaline stress. OsBAG6 also interacted with energy biosynthesis and metabolic pathway proteins that are involved in plant growth and saline-alkaline stress response mechanisms. This study reveals a novel function for mitochondrial localized OsBAG6 proteins in the saline-alkaline stress response alongside OsCaM1-1.

**Supplementary Information:**

The online version contains supplementary material available at 10.1186/s12284-024-00686-z.

## Background

Saline-alkaline soils pose a severe environmental challenge due to their adverse impacts on plant growth. Such soils are globally distributed and are currently estimated to cover an area of 1.1 × 10^9^ hectares (Hossain 2019; Yang et al. 2022); however, the severity and area of impacted soil is increasing due to climate change and poor irrigation and nutrient management practices (Li et al. 2022). Saline-alkaline conditions hinder plant growth via the impacts of ion toxicity, high pH, osmotic stress, and nutrient deficiency (Liu et al. 2021). Saline-alkalization impacts global production by constraining the areas in which many important crops can be cultivated. However, plants have developed a range of morphological, physiological, and molecular responses to cope with saline-alkaline stress, and it is possible that these mechanisms can be exploited to mitigate the impacts of soil degradation (Fang et al. 2021). Although high salt concentrations and high pH often co-occur, most research to date has focused only on salt stress, with relatively few studies examining the combined impact of saline-alkali stress. Rice (*Oryza sativa* L.) is a major cereal crop that feeds more than half the global population. Irrigated cultivation of rice is an effective way to manage saline-alkali conditions (Ma et al. 2022; Zhu et al. 2022) that has produced considerable economic benefits (Zhu et al. 2022). Exploring the molecular mechanisms underpinning saline-alkaline stress tolerance in rice is prominent for the future development of rice varieties with enhanced saline-alkaline tolerance.

B-cell lymphoma 2 (Bcl-2)-associated athanogene (BAG) proteins are found in diverse plant species including *Oryza sativa, Arabidopsis thaliana*, and *Zea mays*, among others (Yan et al. 2003; Doukhanina et al. 2006; Rana et al. 2012; Hu et al. 2013; Zhou et al. 2021). Plant BAG proteins are divided into two classes according to their conserved domains (Rana et al. 2012). Both the class I and class II BAG subfamilies contain a BAG domain. Class I proteins also contain a ubiquitin-like domain that resembles the human BAG1 protein. Class II members contain a calmodulin-binding domain/isoleucine glutamine (IQ motif) that is unique to plants, suggesting possible plant-specific functions (Kabbage and Dickman 2008; Rana et al. 2012; Jiang et al. 2022). The IQ motif can interact with calmodulin (CaM) proteins in the absence or presence of Ca^2+^ in different binding modes (Putkey et al. 2003; Li et al. 2016a; Zhang et al. 2019). *Arabidopsis* AtBAG5, AtBAG6 and AtBAG7 are class II proteins. BAG proteins perform several functions in numerous biological processes, ranging from plant growth and development to biotic stress and abiotic stress responses (Kabbage et al. 2016; Li et al. 2016a; Wang et al. 2020a; Irfan et al. 2021; Zhou et al. 2021). Overexpression of *AtBAG4* in *Arabidopsis* and rice confers salt stress tolerance (Doukhanina et al. 2006; Hoang et al. 2015). Ectopic expression of *AtBAG5* leads to leaf senescence symptoms as a result of CaM/AtBAG5/Hsc70 complex regulation (Li et al. 2016a). AtBAG5 interacts with Ca^2+^-free CaM (Apo-CaM) and Hsc70 using the IQ motif and BAG domain independently, whereas AtBAG5 interacts with Ca^2+^-saturated CaM (Holo-CaM) under a different binding mode that reduces the interaction of AtBAG5 and Hsc70 (Li et al. 2016a). Loss-of-function of *AtBAG7* results in sensitivity to heat stress, and translocation and sumoylation are required for the AtBAG7 heat stress response (Li et al. 2017b). To date, only OsBAG4 has been shown to function in abiotic and biotic stress responses in rice (You et al. 2016; Wang et al. 2020a). However, very few studies have examined OsBAG functions in rice, particularly those of the class II subfamily, and the molecular regulatory mechanisms of OsBAG proteins in subfamily II in rice remain unclear.

Ca^2+^ has been implicated as an essential second messenger involved in the signals transduction from various environmental stimuli to adaptive responses (Tuteja and Mahajan 2007; Ranty et al. 2016). Most proteins binding to Ca^2+^ contain an EF-hand motif, such as CaM, calmodulin-like, Calmodulin B-like protein, and Ca^2+^-dependent protein kinase (CDPK) (Hrabak et al. 2003; DeFalco et al. 2009). Of these, the CaM protein family is one of the most important EF-hand groups, being responsible for transducing increased cytosolic Ca^2+^ signals by interacting and altering the activities of several target proteins, eventually leading to modulate protein interactions and the target genes expression (Boonburapong and Buaboocha 2007). Many diverse CaM target proteins have been identified, including kinases, kinase phosphatases, transcription factors, and metabolic enzymes (Ranty et al. 2006). Unlike other Ca^2+^-sensing proteins, CaM contain no functional domains other than the EF-hand motifs. Five genes encoding CaM proteins have been observed in rice. Among, the amino acid sequence of OsCaM1-1, OsCaM1-2 and OsCaM1-3 are the same, and OsCaM2 and OsCaM3 proteins differing from OsCaM1 by only two amino acids (Boonburapong and Buaboocha 2007). CaM proteins have been implicated in abiotic stress responses, including responses to salt stress and heat stress, in rice (Saeng-ngam et al. 2012; Yuenyong et al. 2018). *OsCam1-1* overexpression led to salt and heat stress tolerance via broad-ranging impacts on the expression of genes participated in multiple cellular processes ranging from hormone-mediated regulation, signaling, transcription, secondary metabolism, to photosynthesis (Saeng-ngam et al. 2012; Wu et al. 2012; Yuenyong et al. 2018). Additional research showed that OsCaM1 was associated with the CCamK-MKK1/6 cascade and positively affected the growth of lateral root under salt stress through auxin signaling (Yang et al. 2021). Further investigation is needed to identify additional binding partners and mechanistic responses involving OsCaM proteins during stress responses.

Here, we found ectopic expression of *OsBAG6* in Kitaake exhibited a dwarf phenotype and produced smaller grains with lower grain weight. Overexpression lines also exhibited elevated saline-alkaline stress-sensitive phenotypes. Loss-of-function mutants (*osbag6*) were identified with enhanced tolerance to saline-alkaline stress, as indicated by increased survival compared with wild-type Kitaake. OsBAG6 localized to mitochondria. The expression level of *OsBAG6* was induced by several abiotic stresses including saline, saline-alkaline, heat and abscisic acid (ABA) treatment, which detected by Real-time quantitative PCR (RT-qPCR). Under normal conditions, OsBAG6 interacts with OsCaM1-1. However, the increase in Ca^2+^ concentration within the cytoplasm under saline-alkaline stress, and consequent increased Ca^2+^-OsCaM1-1 binding, led to dissociation of OsCaM1-1 from OsBAG6. This activated the expression of genes downstream of OsCaM1-1 that functioned in saline-alkaline stress response. These studies provide fresh insights into the function of rice OsBAG6 in plant growth and during responses to saline-alkaline stress.

## Materials and Methods

### Plant Materials and Growth Conditions

CRISPR (Clustered Regularly Interspaced Short Palindromic Repeats)/Cas9 technology was used to generate *osbag6* mutants according to the previously described method (Lu et al. 2017; Nan et al. 2020). All loss-of-function mutants and transgenic plants used in this study were in the Kitaake (japonica variety) background. For physiological analysis, seeds were sterilized for 20 min in sodium hypochlorite solution and washed 4 times before being immersed in distilled water for 3 d at 37 °C. Germinated seeds were cultured in Yoshida’s culture solution in a bottomless 96-well plate (Yoshida 1976). Four-week-old seedlings were placed into Yoshida’s nutrient solution containing 25 mM Na_2_CO_3_ (pH = 10.0) for 5 d treatment and then subject to normal nutrient culture solution for 3 d recovery (Liu et al. 2021). The EC value of 25 mM Na_2_CO_3_ at 25 °C ≈ 4.74 mS/cm, which was measured by an EC meter (METTLER TOLEDO, Switzerland). Survival rates were calculated according to the percentage of alive seedlings (plants with green leaves), 32 plants in each genotype were used to calculate survival rates, and 5 biological replicates were performed. Unless described specifically, all physiological analyses were grown in a phytotron (200 μM photons m^−2^ s^−1^ light intensity, 14 h light (28 °C)/10 h dark (25 °C)) at ~ 70% relative humidity.

### Plasmid Construction

To generate *OsBAG6* overexpression lines (*OsBAG6OE*), the coding sequence (CDS) of *OsBAG6* was cloned using cDNA as the template by primers OsBAG6-F/R into *pCsV1300* in-frame with the 2 × FLAG using *Xba*I and *Eco*RI. In order to obtain loss-of-function mutants (*osbag6*), we generated the corresponding construct using primer pairs OsBAG6-CRISPR-F/R cloned into *pYLsgRNA-OsU6a* and *pYLCRISPR/Cas9P*_*Ubi*_*-H* plasmid (Ma and Liu 2016; Xie et al. 2017). We generated the *OsBAG6-GFP* plasmid to examine the subcellular localization, the CDS of *OsBAG6* was cloned in-frame with GFP under the control of cauliflower mosaic virus (CaMV) 35S promoter. To generate *OsBAG6* complementation lines, *OsBAG6*_*pro*_*:OsBAG6-2* × *FLAG* was constructed in *pCAMBIA1302* binary vector. The promoter of *OsBAG6* (1529 bp fragment upstream of *OsBAG6* start codon) was cloned using OsBAG6-pro-F/R. For co-IP assay, the CDS of *OsBAG6* and *OsCaM1-1* were cloned into vectors *326-HA* and *326-FLAG* using a CloneExpress Ultra One Step Cloning kit (C115; Vazyme, Nanjing, China) (Wang et al. 2020a). The CDS of *OsCaM1-1* was obtained from cDNA of Kitaake using primer pairs OsCaM1-1-F/R. For protein extraction, *GST-OsCaM1-1* was generated by cloning corresponding fragments into vector *pGEX4T-1* (Invitrogen, Carlsbad, CA, USA). OsBAG6-FLAG was also fused to 3′ end of MBP in vector *pMAL-c6T* (New England Biolabs [NEB], Ipswich, MA, USA) to purify proteins. To investigate the interaction using yeast two-hybrid (Y2H), the CDS of *OsBAG6* was cloned using primer pairs OsBAG6-AD-F/R into *pGADT7* to construct *AD-OsBAG6*. The CDS of *OsCaM1-1* was cloned using OsCAM1-1-BD-F/R into *pGBKT7* in-frame with a DNA-binding domain of GAL4 (BD). Additional file 2: Table S1 listed all used primers, and all constructs were sequenced for confirming.

### Generation of Transgenic Plants

*Agrobacterium*-mediated transformation was used to generate *osbag6* mutants by introducing into the corresponding constructs. The transgenic lines were selected by hygromycin (50 mg L^−1^) and the mutation forms in *OsBAG6* loci of *osbag6* mutant were confirmed by Sanger sequencing. To generate *OsBAG6* complementation lines and *OsBAG6OE* transgenic plants, *OsBAG6*_*pro*_*:OsBAG6-2* × *FLAG* or *CsV*_*pro*_*:OsBAG6-2* × *FLAG* was introduced into *osbag6-1* or Kitaake background, and western blot were used to verify the OsBAG6-2 × FLAG proteins. T3 homozygous were identified by applying hygromycin (50 mg L^−1^) (Chen et al. 1998).

### Subcellular Localization Analysis

Subcellular localization analysis was performed as previously described (Wang et al. 2020a). Leaf sheath of 3-week-old seedlings were used to isolate protoplast. Using polyethylene glycol-mediated transfection, *OsBAG6-GFP* plasmid and *OsCOX11*-*mCherry* plasmid were co-transfected to protoplast for 12–16 h culturing before observation (Zhang et al. 2011; Han et al. 2021, 2022). A fluorescence microscope (Olympus BX53, Japan) was used to observe cells expressing fluorescently tagged fusion proteins. Wavelengths used for excitation and emission of GFP and mCherry were accomplished by a GFP filter set (Excitation = 480/20 nm, Emission = 510 nm, Dichroic Mirror = 505 nm) and an RFP filter set (Excitation = 535/30 nm, Emission = 580 nm, Dichroic Mirror = 565 nm). Images were obtained and processed using QCapture Pro 7 Software (Teledyne Photometrics).

### RNA Isolation and RT-qPCR Analysis

To detect the expression pattern of *OsBAG6*, two-week-old Kitaake seedlings were treated with 200 mM NaCl, 25 mM Na_2_CO_3_, 15% PEG 4000, 4 °C, 45 °C, and 0.1 mM ABA. Three whole seedlings were pooled as a sample to isolate high-quality total RNA with the TRIzol reagent (Invitrogen, Carlsbad, CA, USA). cDNA Synthesis SuperMix (TransGen Biotech, Beijing, China) was used to conduct reverse transcription reaction. RT-qPCR was performed using the Applied Biosystems QuantStudio 3 instrument (ThermoFisher, Waltham, MA, USA) with the SYBR green (TOYOBO, Shanghai, China) as described previously (Wang et al. 2020a). As an internal control, Rice *OsGAPDH1* (OsKitaake08g018600) was used, and results were calculated using 2^−△△CT^ method. Primers used in RT-qPCR assay are listed in Additional file 2: Table S1.

### RNA-Seq Analysis

The roots and shoots of 3-week-old Kitaake and *OsBAG6OE* seedlings were used for total RNA extraction with TRIzol reagent, 5 seedlings pooled together as a sample. Three independent biological replicates were performed for each genotype. Briefly, 3 μg total RNA from each sample was prepared to construct library. Sequencing was accomplished by MGISEQ-2000 (BGI, Shenzhen, China). Data was analyzed by FASTX-Toolkit (vision 0.0.13), HISAT2 (vision 2.1.0), and CUFFLINKS (http://cole-trapnell-lab.github.io/cufflinks/cuffdiff/index.html). *Oryza sativa* Kitaake (JGI v3.0) gene annotation as the transcript index. The homolog of DEGs in Kitaake was found in Nipponbare (*NIP*), and GO analysis were used the go term constructed based on *NIP* genome.

### DAB Staining Assay

As previously reported, the diaminobenzidine (DAB) staining was performed (Nan et al. 2020). Briefly, 4-week-old seedlings’ leaves without or with 24 h 25 mM Na_2_CO_3_ (pH = 10.0) treatment were used for staining. The leaves of indicated genotypes were stained at room temperature in 0.1% DAB-tetrahydrochloride (pH = 5.8) for 24 h after vacuum-infiltrated for 1 h. Leaves were mounted in buffer (ethanol:glycerol:lactic acid = 3:1:1) at 80–90 °C until colorless, and then immersed in 70% ethanol.

### Metal Content Determination

Shoots and roots of seedlings before and after 5-d 25 mM Na_2_CO_3_ treatment were harvested and washed 5 times using deionized water before oven-dried to reach a constant mass at 55 °C (At the initial stage, 105 °C was used for 10 min). 200 mg of each dry sample was powdered, followed by digestion with 2 mL nitric acid using a heat block. The ion content of Fe, Mn, and Zn was determined by inductively coupled plasma mass spectrometry (iCAP RQ, Thermo Scientific, USA), and the Na^+^ concentration was determined by inductively coupled plasma optical emission spectrometer (LEEMAN Prodigy, USA).

### Immunoprecipitation–Mass Spectrometry (IP-MS)

The IP-MS was performed as previously described (Yang et al. 2021). In general, 2 g of total plant from the wild-type Kitaake and two independent transgenic lines carrying *CsV*_*pro*_*:OsBAG6-2* × *FLAG* were collected and ground in liquid nitrogen and then homogenized in 30 mL IP buffer (2 mM DTT, 150 mM NaCl, 100 mM Tris–HCl [pH = 7.5], 5 mM EGTA, 0.5% Triton X-100, 5 mM EDTA, 1 × Protease Inhibitor Cocktail [Roche, Basel, Switzerland]). Total proteins jointly incubate with anti-FLAG M2 magnetic beads (M8823; Sigma, USA) at 4 °C for 3 h. Beads-bound proteins were further analyzed by Suzhou Mass-elife biotechnologies Co., Ltd (Suzhou, China). *Oryza sativa* MSU release 7 rice gene annotation was used as the background. The orthologs in Kitaake were found.

### Co-immunoprecipitation (co-IP)

Rice protoplasts were transfected with OsBAG6-FLAG construct and OsCaM1-1-HA constructs. After incubation for 14 h, the protoplasts were harvested and suspended in IP buffer. The samples were sonicated and centrifugated, then supernatant was used to incubate with magnetic beads attaching anti-FLAG antibody (M8823; Sigma, USA) after taking out the input. The samples were rotated for 3 h at 4 °C. The magnetic beads were harvested and washed 4 times before sampling. Anti-FLAG antibody (F7425; Sigma, USA) and anti-HA antibody (H6908; Sigma, USA) were used for protein detection.

### Purification of Recombinant Protein

The assay was performed as previously described with slight modifications (Liu et al. 2022). In brief, *Escherichia coli* BL21 (DE3) strains were used to express fusion constructs including *GST-OsCaM1-1* and *MBP-OsBAG6-FLAG* at 25 °C for 6 h by adding 0.5 mM IPTG (isopropyl β-D-1-thiogalactopyranoside). The supernatant was discarded after the bacterial cultures collecting, and lysis buffer (1 × Protease Inhibitor Cocktail [Roche, Switzerland], 0.1% Triton X-100, 2 mM DTT, in PBS buffer) was added to the precipitate for resuspending. Glutathione–Sepharose beads (10250335; GE Healthcare, Uppsala, Sweden) or Amylose resin (E8021, NEB, USA) were used to purify the recombinant fusion proteins. The purified protein was confirmed by SDS-PAGE and prepared for pull-down assay (Xu et al. 2013).

### Pull-Down Assay

To detect the binding affinity between OsBAG6 with OsCaM1-1 in the absence or presence of Ca^2+^, 1 μg MBP-OsBAG6-FLAG and 2 μg GST-OsCaM1-1 were incubated with anti-FLAG M2 magnetic beads (M8823; Sigma, USA) in buffer (1 mM DTT, 0.5% Triton X-100, MG132, 150 mM NaCl, 100 mM Tris–HCl [pH = 7.5], and 1 × Protease Inhibitor Cocktail), the systems were added by 5 mM EGTA or 2 mM CaCl_2_. The beads were washed 4 times after 3 h incubation, and subjected to sample preparation to perform Western blot.

### Immunoblotting

To detect GST epitope, anti-GST (HT601-01) antibody was obtained from TransGen Biotech (Beijing, China). As an internal control, anti-H3 antibody (Ab1791; Cambridge, MA, USA) was obtained from Abcam. ECL Plus Western Blotting Detection System was used to detect the western blots (MA0186; Meilunbio, Dalian, China).

### Yeast Two-Hybrid Assay

Matchmaker™ Gold Yeast Two-Hybrid System was used to perform yeast two-hybrid assay (Clontech, San Jose, CA, USA). In brief, the full-length CDS of *OsBAG6* was cloned into vector *pGADT7* in frame with the GAL4 activation domain (AD). The *OsCaM1-1* was cloned into vector *pGBKT7*, which harbors a GAL4 DNA-binding domain (BD). AD constructs were transformed into Y187, and BD constructs were transformed into Y2H Gold yeast strains, respectively. Synthetic dropout (SD) medium (SD/-Trp/-Leu/-His/-Ade+X-α-Gal or SD/-Trp/-Leu) were used to selected the mating yeast (Wang et al. 2020b).

### Phylogenetic Analysis by Maximum Likelihood Method

MEGA X was used to build phylogenetic tree with the Maximum Likelihood method (1000 bootstrap replications) and Poisson correction model (Kumar et al. 2018). Below the branches, the percentage of trees in which the associated taxa clustered together is shown. The phylogenetic tree is drawn to scale, with branch lengths measured in the number of substitutions per site.

## Results

### Phylogenetic Analysis of BAG6 Orthologs in Dicots and Monocots

**Fig. 1:**
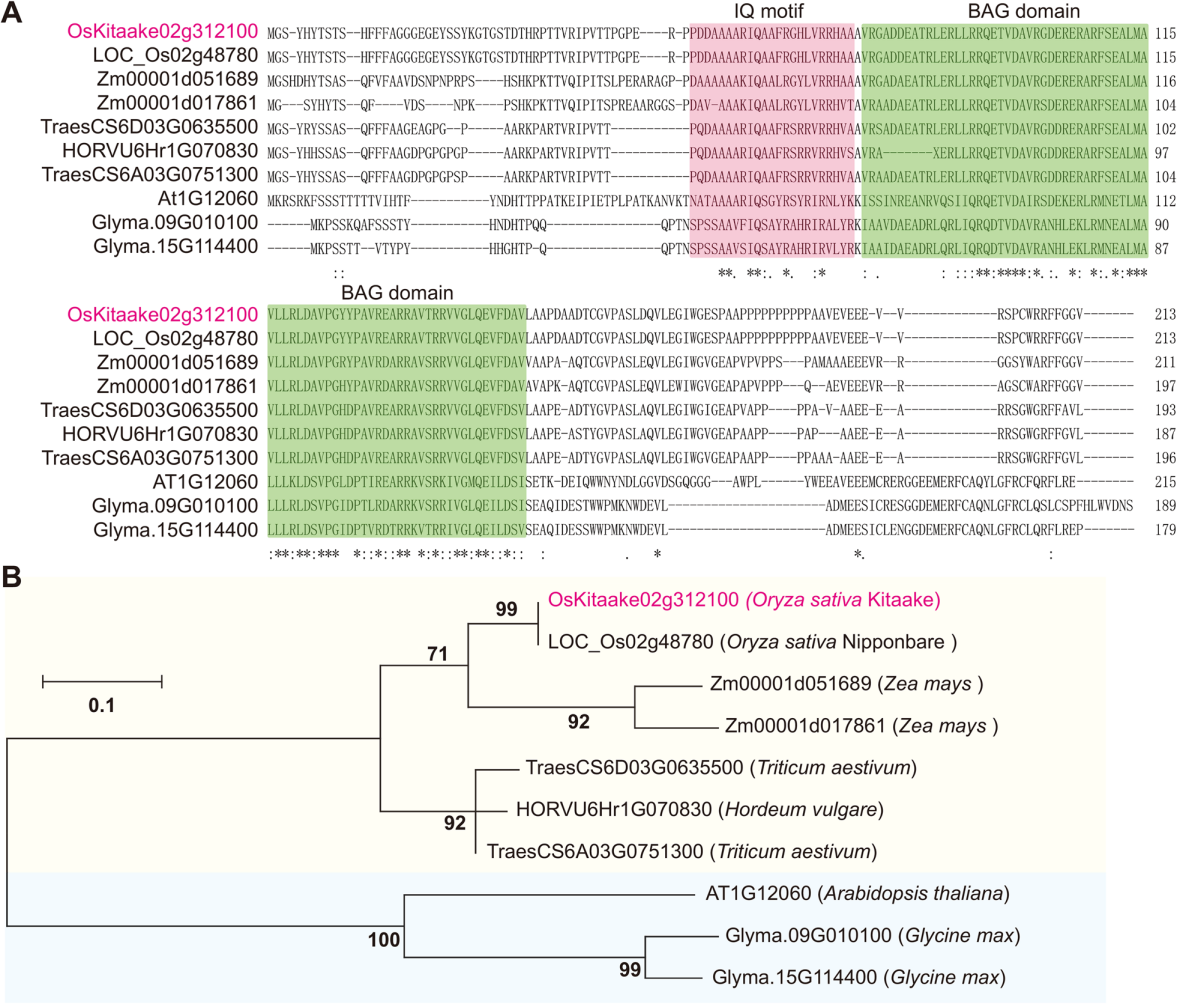
Multiple alignment of amino acid sequence and phylogenetic analysis of BAG6 orthologs in plants. **A** Multiple alignment of amino acid sequence of OsBAG6 and its orthologs in other species. The IQ motif and BAG domain are shaded in different colors. **B** The phylogenetic tree was constructed by Maximum Likelihood method using MEGA X software using the amino acid sequences of the proteins. The sequences of the proteins were obtained from Phytozome 13 (https://phytozome-next.jgi.doe.gov/). The tree is drawn to scale, with branch lengths measured in the number of substitutions per site. Monocots or dicotyledons are shaded in yellow or blue colors, respectively

Bioinformatic prediction revealed that OsBAG6 has the smallest molecular weight among the identified BAG proteins (Wang et al. 2020a). To isolate potential Kitaake OsBAG6 orthologs in plant species, the amino acid sequence of OsBAG6 was used for BLASTp search (Expect [E] threshold < − 2) in the *Oryza sativa* Nipponbare, *Triticum aestivum*, *Zea mays*, *Hordeum vulgare*, *Arabidopsis thaliana*, and *Glycine max* genomes using Phytozome (https://phytozome-next.jgi.doe.gov/). Multiple alignment analysis displayed that BAG6 orthologs from rice, maize, wheat, barley, soybean and *Arabidopsis* all contained an IQ motif and BAG domain (Fig. 1A). Phylogenetic analysis further revealed that monocotyledon and dicotyledon BAG6 proteins formed separate discrete clusters (Fig. 1B), highlighting the relatively large sequence differences and long differentiation time between monocotyledons and dicotyledons and suggesting that BAG6 functions may differ between monocotyledons and dicotyledons.

### Expression Patterns and Subcellular Localization of *OsBAG6*

**Fig. 2:**
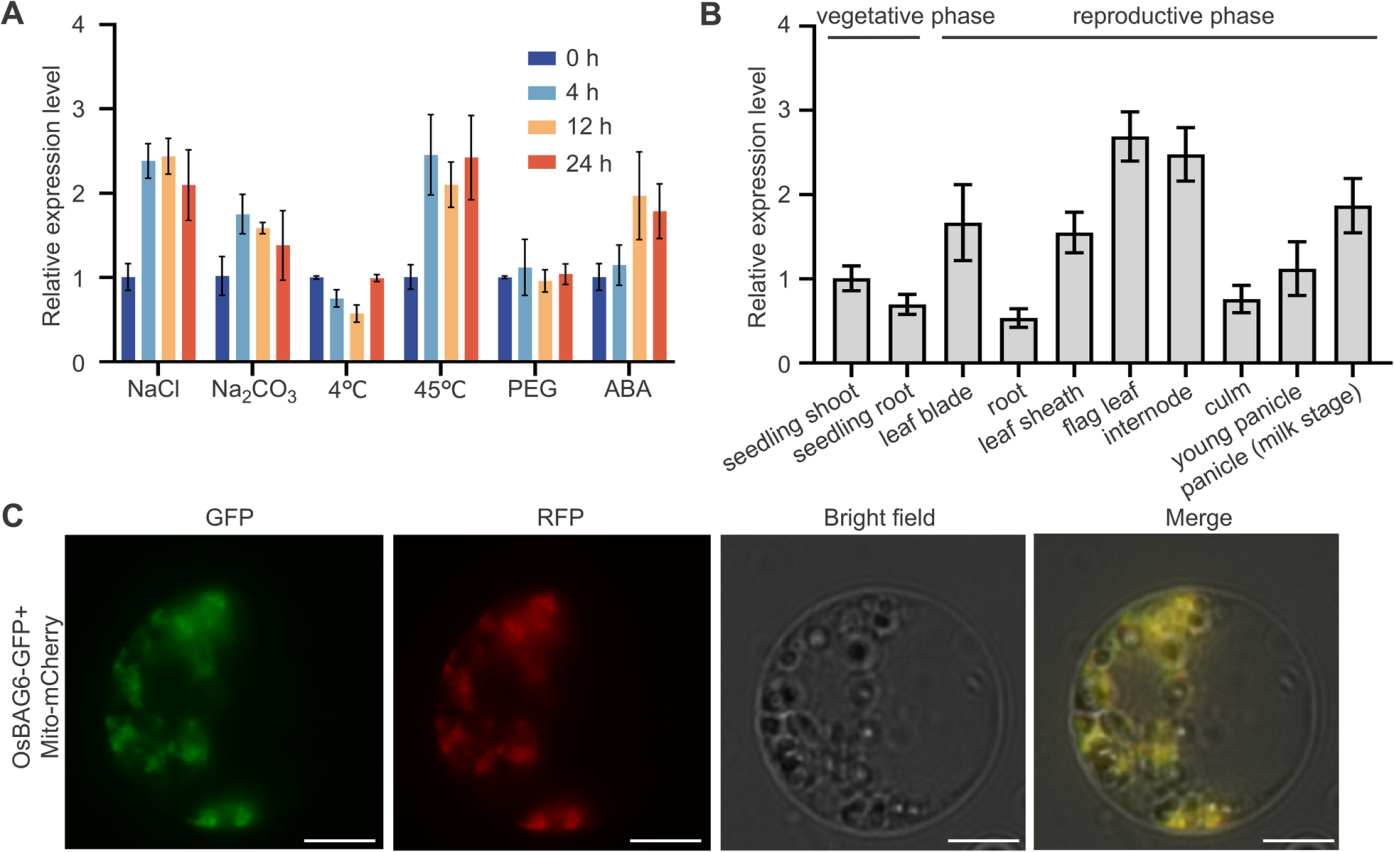
Subcellular localization and spatial and temporal expression patterns of *OsBAG6*. **A** Expression patterns of *OsBAG6* under abiotic stress and ABA treatment. *OsGAPDH1* was used as the internal control. Error bars indicate ± SD (*n* = 3). **B** Relative expression level of *OsBAG6* in the indicated tissues of Kitaake plants, as determined by real-time quantitative PCR (RT-qPCR). *OsGAPDH1* was used as the internal control. Error bars indicate ± SD (*n* = 3). **C** Subcellular localization analysis of OsBAG6. *OsBAG6-GFP* was co-transfected with *OsCOX11*-*mCherry* plasmid into rice (Kitaake) protoplasts. GFP, green fluorescent protein; RFP, red fluorescent protein. Scale bar = 10 μm

Previous research showed that BAG proteins played roles in abiotic stress responses (Li et al. 2017b; Wang et al. 2020a). Here, *OsBAG6* expression was examined in Kitaake treated with 200 mM NaCl, 25 mM Na_2_CO_3_, 4 °C, 45 °C, or 15% PEG to induce salt, saline-alkaline, cold, high-temperature, and osmotic stress responses, respectively. Kitaake seedlings were also treated for 0, 4, 12, and 24 h with 0.1 mM ABA. *OsBAG6* expression was significantly elevated under NaCl and 45 °C treatments, and slightly elevated after Na_2_CO_3_ treatment and 12 h ABA treatment (Fig. 2A). Upon exposure to cold stress, *OsBAG6* expression initially decreased and then subsequently increased to base levels after 24 h (Fig. 2A). To examine the tissue-specific expression level of *OsBAG6*, the RNA was isolated from vegetative and reproductive Kitaake tissues and used in real-time quantitative PCR (RT-qPCR) to explore spatial and temporal expression patterns of *OsBAG6*. As shown in Fig. 2B, *OsBAG6* was expressed in shoots and roots in the vegetative phase in 4-week-old seedlings and was also abundantly expressed in flag leaves and the internode. Relative high expression levels were also observed in reproductive phase tissues (leaf blade, leaf sheath, and mature panicle at the milk stage). Low expression levels were observed in roots in both mature vegetative and reproductive tissues (Fig. 2B). Next, to examine the subcellular localization of OsBAG6, a plasmid expressing *35S*_*pro*_*:OsBAG6-GFP* was generated. The construct was co-transfected with *OsCOX11-mCherry*, a previously described mitochondrial localization protein marker (Luo et al. 2013; Han et al. 2021, 2022). As shown in Fig. 2C, OsBAG6-GFP co-localized with OsCOX11-mCherry, demonstrating mitochondrial localization of OsBAG6. Overall, these results indicate that OsBAG6 localizes to the mitochondria and is expressed in both vegetative and reproductive stages as well as after exposure to salt, saline-alkaline, and heat stresses.

### *OsBAG6* Acts as a Negative Regulator of Saline-Alkaline Stress Tolerance

**Fig. 3:**
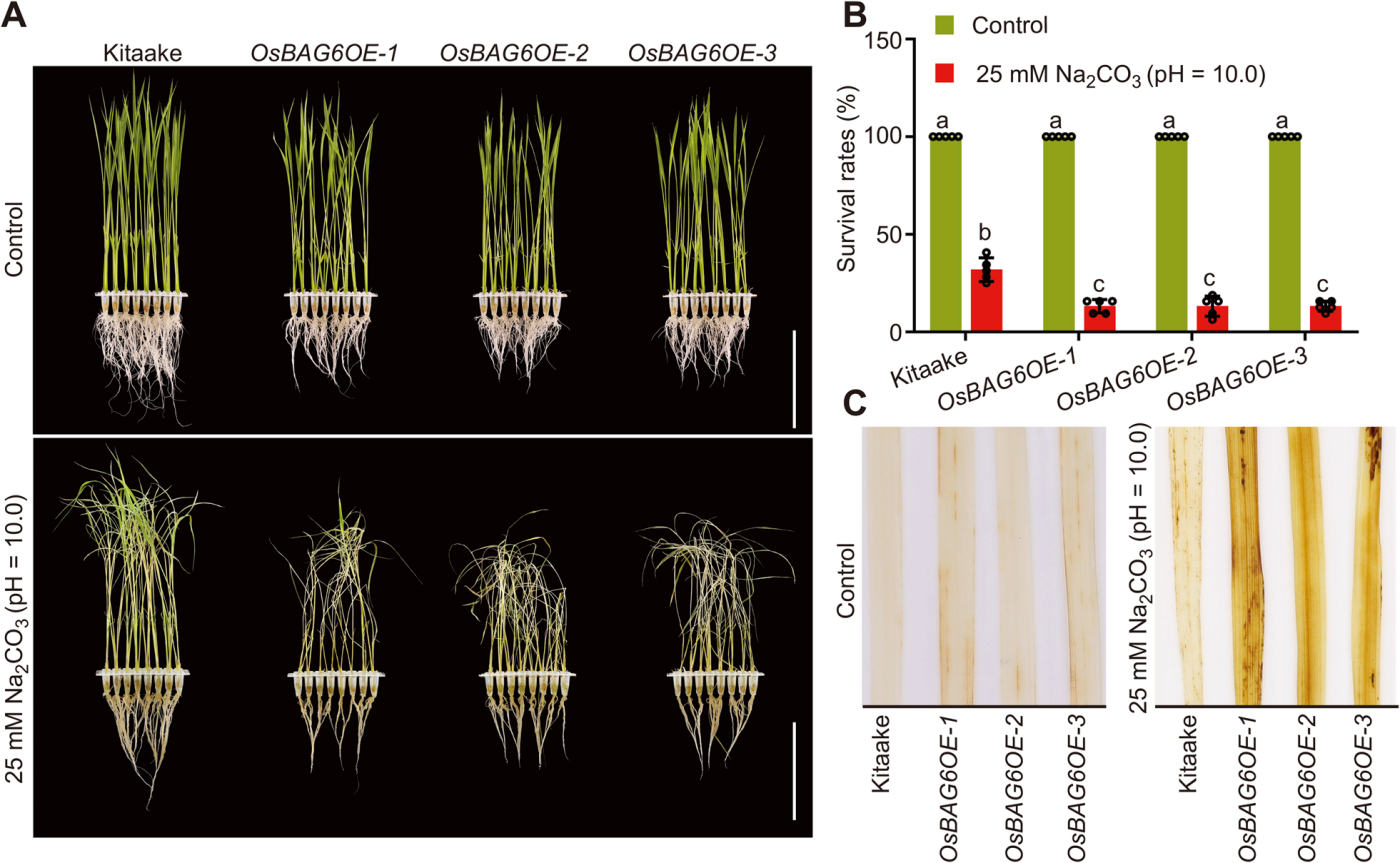
Characterization of *OsBAG6* overexpression lines under saline-alkaline stress conditions. Image (**A**) and survival rates (**B**) of Kitaake and 3 independent *OsBAG6OE* lines before and after recovery from saline-alkaline stress condition (25 mM Na_2_CO_3_, pH = 10.0). In **A**, scale bar = 8 cm. In **B**, Data represent mean ± SD of 5 replicates (*n* = 5, 32 plants per genotype were used to calculate survival rate per replicate). Significant differences were evaluated by two-way ANOVA, followed by Tukey’s multiple comparison test. **C** DAB staining of leaves of indicated genotypes before and after 24 h saline-alkaline stress (25 mM Na_2_CO_3_, pH = 10.0). Three biological replicates were performed, with 25 plants per treatment

To investigate the function of OsBAG6, *OsBAG6-*overexpressing transgenic lines were constructed. The *CsV*_*pro*_*:OsBAG6-2* × *FLAG* construct was introduced to wild-type (WT) Kitaake (Additional file 1: Fig. S1A), and three independent homozygous *OsBAG6-*overexpressing lines (*OsBAG6OE-1*, *OsBAG6OE-2* and *OsBAG6OE-3*) were generated. Western blot analysis was performed to examine the production of OsBAG6-FLAG protein in all three lines with expected sizes (Additional file 1: Fig. S1B). Subsequently, *OsBAG6OE* lines and Kitaake plants were exposed to saline-alkaline stress treatment (25 mM Na_2_CO_3_, pH = 10.0). *OsBAG6OE* plants showed strong saline-alkaline stress-sensitive phenotypes compared with WT in terms of survival rates (Fig. 3A, B). Production of H_2_O_2_ was examined in transgenic and Kitaake leaves under normal conditions and after 24 h saline-alkaline treatment by staining with diaminobenzidine (DAB). Under normal conditions, no significant differences in H_2_O_2_ levels between *OsBAG6OE* and Kitaake were detected, but transgenic lines accumulated more H_2_O_2_ than Kitaake under saline-alkaline stress treatment (Fig. 3C).

**Fig. 4:**
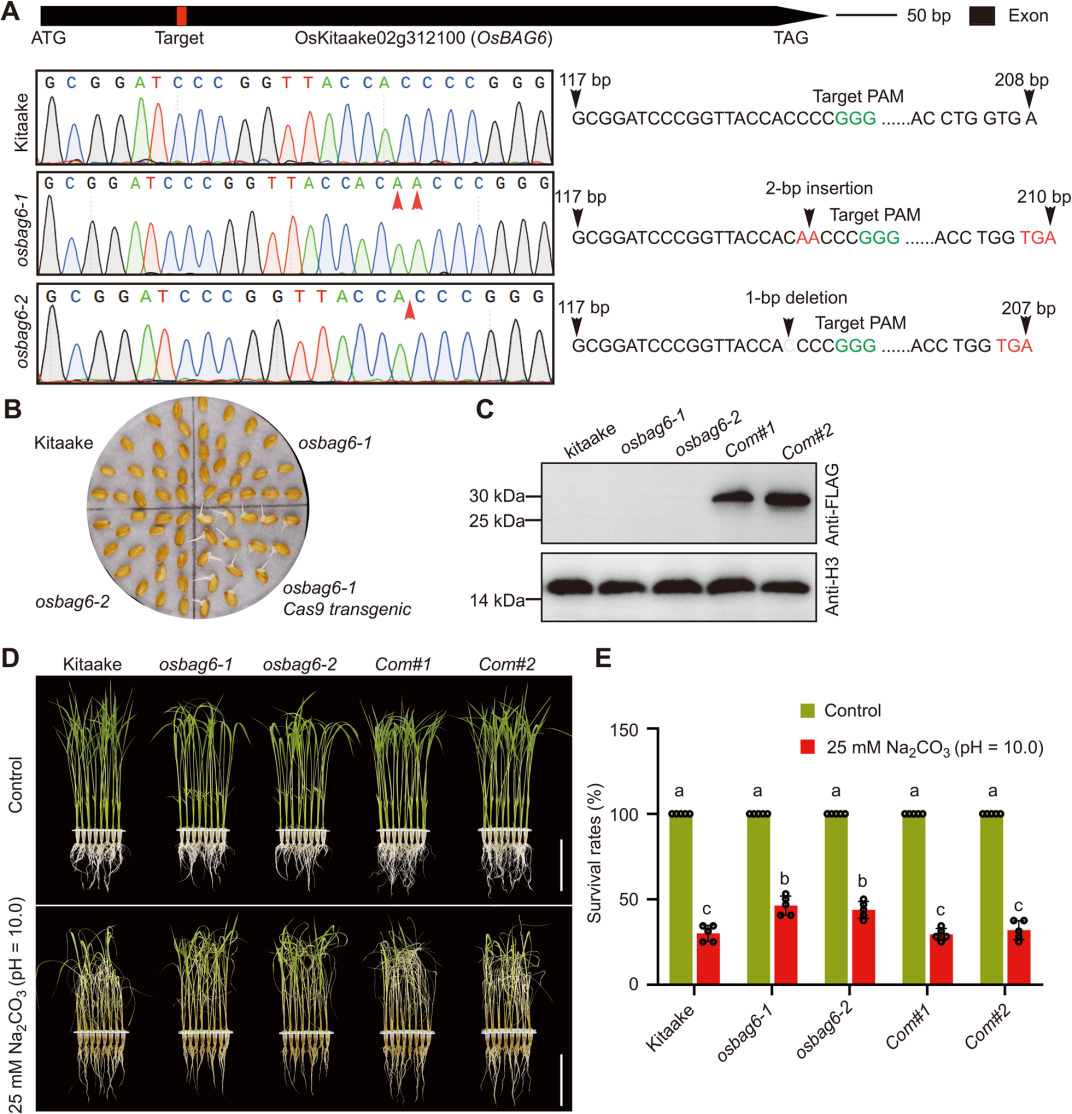
Characterization of *osbag6* mutant lines under saline-alkaline stress conditions. **A** Sequence chromatograms showing mutations introduced in the *OsBAG6* gene in the *osbag6-1* and *osbag6-2* mutants using CRISPR/Cas9 technology, as revealed by Sanger sequencing. **B** Isolation of Cas9-free mutants. The indicated genotypes were sown in media including 50 mg L^−1^ hygromycin. **C** Immunoblot analysis of OsBAG6-FLAG in Kitaake, *osbag6-1*, *osbag6-2*, and 2 independent complementation lines (*Com#1* and *Com#2*) using anti-FLAG antibody. Histone H3 was used as a loading control. Image (**D**) and survival rate (**E**) of Kitaake, 2 independent *osbag6* mutant lines, *Com#1* and *Com#2* before and after recovery from saline-alkaline stress condition (25 mM Na_2_CO_3_, pH = 10.0). In **D**, scale bar = 8 cm. In **E**, Data represent mean ± SD of 5 replicates (*n* = 5, 32 plants per genotype were used to calculate survival rate per replicate). Significant differences were evaluated by two-way ANOVA, followed by Tukey’s multiple comparison test

To further characterize the role of *OsBAG6* in saline-alkaline stress tolerance, two independent *OsBAG6* knock-out mutants (*osbag6-1* and *osbag6-2*) were generated. Single guide RNA (sgRNA) target sites for *OsBAG6* were cloned into a CRISPR/Cas9 vector in which *Cas9* was driven by the maize *Ubiquitin10* promoter (Ma et al. 2015). Two homozygous mutant lines (*osbag6-1* and *osbag6-2*) were generated in the Kitaake background and verified by Sanger sequencing. The *osbag6-1* mutant carried a 2-bp insertion located 134 bp downstream of the ATG start codon, and the *osbag6-2* mutant carried a 1-bp deletion at the same location. Both mutations produced a frameshift before the IQ motif and generated a premature stop codon (Fig. 4A). To exclude the potential effect of the *Cas9* gene, *osbag6-1* and *osbag6-2* mutant plants were screened for hygromycin sensitivity to select mutants lacking *Cas9* (Fig. 4B). To confirm the *osbag6* mutant phenotype was due to loss of *OsBAG6*, complementation lines were generated by transfecting FLAG epitope-tagged *OsBAG6* under the control of *OsBAG6* promoter (*OsBAG6*_*pro*_*:OsBAG6-2* × *FLAG*) into the *osbag6-1* mutant. OsBAG6-FLAG protein production was confirmed by western blot analysis in 2 independent complementation lines (*Com#1* and *Com#2*) (Fig. 4C). The two loss-of-function mutant lines displayed enhanced saline-alkaline stress tolerance, and the complementation lines displayed survival rates similar to those of Kitaake plants under saline-alkaline stress treatment (Fig. 4D, E).

**Fig. 5:**
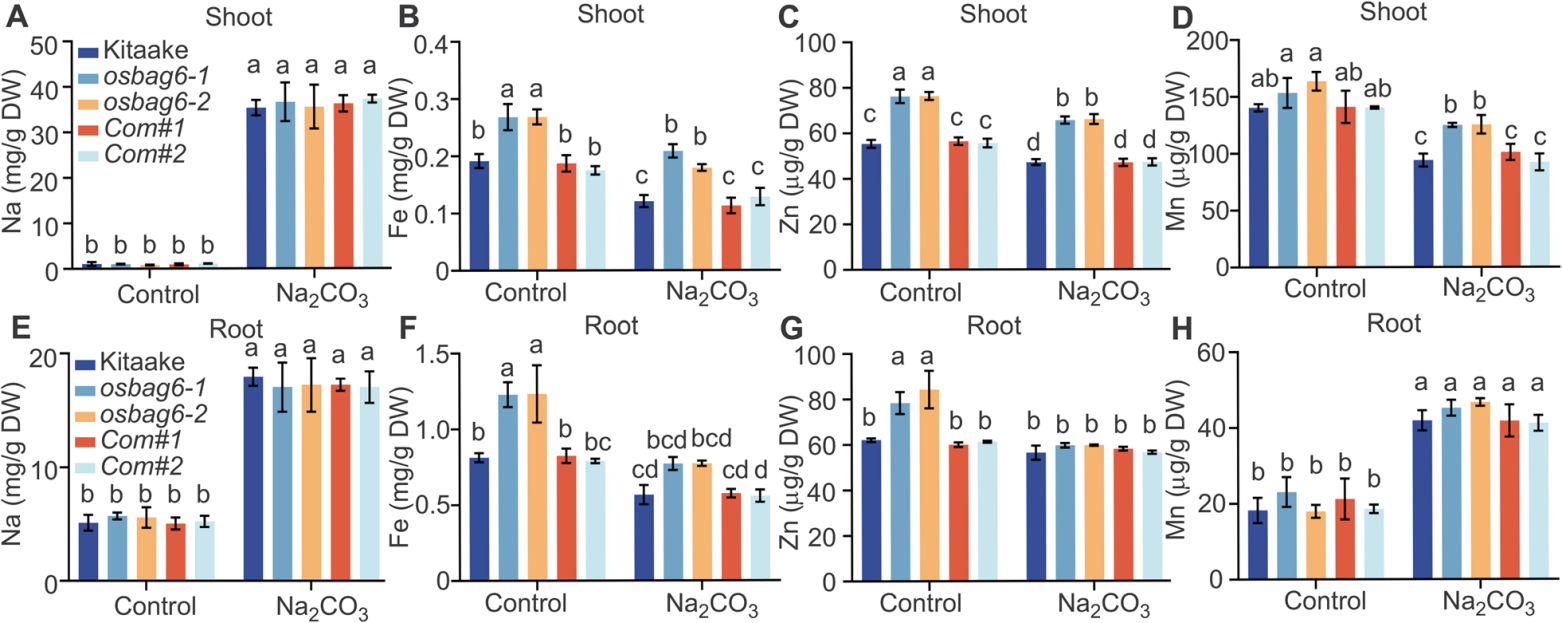
Effect of saline-alkaline stress on Na, Fe, Zn, and Mn in the shoots and roots. **A**–**D** The concentration of Na, Fe, Zn, and Mn in the shoots of kitaake, *osbag6* mutants and *OsBAG6* complementation lines under normal and 25 mM Na_2_CO_3_ treatment conditions. **E**–**H** The concentration of Na, Fe, Zn, and Mn in the roots of kitaake, *osbag6* mutants and *OsBAG6* complementation lines under normal and 25 mM Na_2_CO_3_ treatment conditions. Data represent mean ± SD of 3 replicates (20 plants per genotype were used for sampling). Significant differences were evaluated by two-way ANOVA

Rice tends to take up significant quantities of Na^+^ while impeding the absorption of other essential nutrients like K^+^ under saline-alkaline stress, leading to an imbalance of ions and resulting toxicity (Wang et al. 2018). Additionally, alkaline stress typically restricts the bioavailability of iron (Fe), zinc (Zn), and manganese (Mn) (Aloo et al. 2023). Under saline-alkaline stress, the concentration of Fe in both the roots and shoots of rice significantly decreased (Li et al. 2016b), while that of Mn and Zn decreased in the shoots and Mn increased in the roots (Nampei et al. 2021). Hence, we measured the concentration of sodium (Na), Fe, Mn, and Zn under normal and saline-alkaline stress conditions in kitaake, *osbag6* mutants, and *OsBAG6* complementation lines. As shown in Fig. 5A, no significant differences in Na ion concentrations were observed in the shoots between the wild type and the *osbag6* mutants under the normal and saline-alkaline stress conditions. However, under the normal condition, higher concentrations of Fe and Zn ions were found in the *osbag6* mutant compared with those in kitaake (Fig. 5B, C). Additionally, once exposed to saline-alkaline stress, the concentrations of Fe, Mn, and Zn in the shoots of kitaake and *osbag6* mutants significantly decreased (Fig. 5B–D). The concentrations of Fe, Mn, and Zn concentration still be higher in *osbag6* mutants under saline-alkaline stress (Fig. 5B–D). In the roots, under the normal condition, the concentrations of Na and Mn ions were similar among the various genotypes, while those of Fe and Zn were higher in the *osbag6* mutant than in kitaake (Fig. 5E–H). The concentrations of Na and Mn increased in all genotypes under saline-alkaline stress (Fig. 5E, H). Fe was decreased in the roots of various genotypes and Zn was decreased in the *osbag6* mutant under saline-alkaline stress treatment (Fig. 5F, G). Overall, these results may indicate that OsBAG6 participates in responding to saline-alkaline stress through regulating the homeostasis of Fe, Mn, and Zn.

Additionally, we conducted soil-based experiments to characterize the phenotypes of *OsBAG6OE* and *osbag6* under saline-alkaline stress. Different genotypes were planted in a soil-based pot, and seedlings grown for 2 weeks were irrigated with 25 mM Na_2_CO_3_ (dissolved in water, pH ≈ 11.2). It was found that, after 7 days of treatment, there was no significant difference in terms of withered leaves and dead seedlings (Additional file 1: Fig. S2A, B). However, the wild-type plants exhibited greater height compared to the *OsBAG6OE* plants (Additional file 1: Fig. S2A). After another 17-d treatment (24-d treatment), the *osbag6* mutant plants showed significantly better growth compared to the Kitaake and *OsBAG6* complementation lines, while the *OsBAG6OE* lines had a low survival rate (Additional file 1: Fig. S2A–D). When subjected to an additional 2 weeks (38-d treatment) of saline-alkaline stress, nearly 90% of the *OsBAG6OE* seedlings died, the survival rates of Kitaake still were higher than that of *OsBAG6OE* plants and the survival rates of *osbag6* mutants were higher than that in Kitaake (Additional file 1: Fig. S2A–D). Furthermore, we also test the phenotypes of *OsBAG6OE* plants and *osbag6* mutants under salt stress conditions. As shown in Additional file 1: Fig. S3, we found that *OsBAG6OE* lines exhibited salt stress-sensitive phenotypes, and the *osbag6* mutants exhibited slightly increased salt stress tolerance phenotypes compared with Kitaake, which consisted of saline-alkaline stress. Overall, these results indicate that *OsBAG6* plays a negative role in saline-alkaline stress tolerance.

### Overexpression of *OsBAG6* Negatively Affects Plant Height, Grain Size, and Grain Weight

**Fig. 6:**
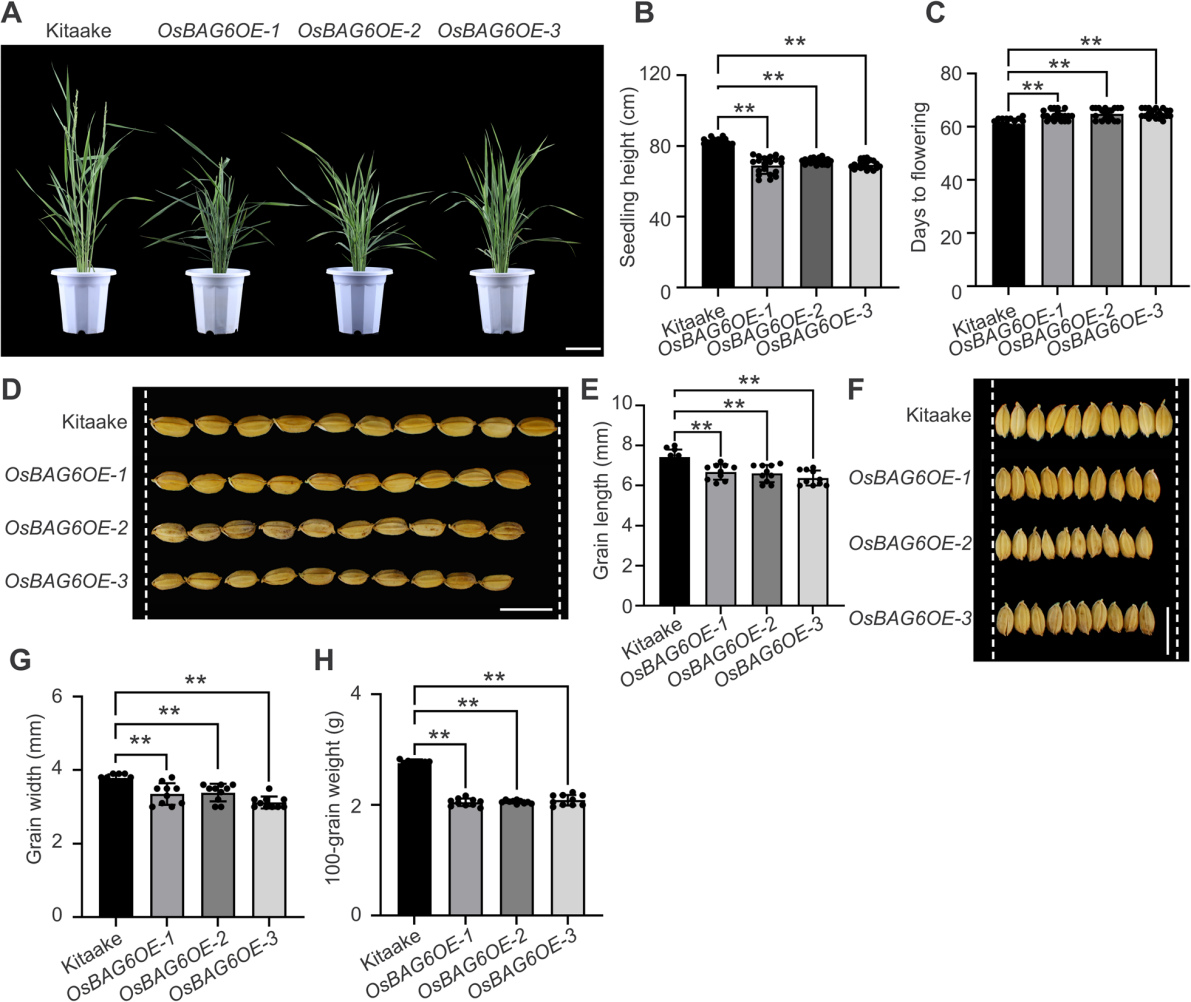
Agronomic traits of *OsBAG6* overexpression (*OsBAG6OE*) lines. **A** Photographs of 65-day-old Kitaake, *OsBAG6OE-1*, *OsBAG6OE-2* and *OsBAG6OE-3* plants in a green house. Scale bar = 13 cm. **B** Plant height of the indicated genotypes. Data represent mean ± SD (*n* = 20 plants per genotype). **C** Heading date of the indicated genotypes. Data represent mean ± SD (*n* = 20 plants per genotype). Image (**D**) and measurements (**E**) of grain length in the indicated genotypes. Scale bar = 1 cm, Data represent mean ± SD of 10 biological replicates. Image (**F**) and measurements (**G**) of grain width in the indicated genotypes. Scale bar = 1 cm, Data represent mean ± SD of 10 biological replicates. **H** Measurements of 100-grain weight in the indicated genotypes. Data represent mean ± SD of 10 biological replicates (each contains 100 grains). Significant differences were evaluated by one-way ANOVA, followed by Tukey’s multiple comparison test, ** represent *p* < 0.01

As shown in Fig. 3A, *OsBAG6OE* transgenic plants exhibited relative shorter plant height compared with Kitaake in seedling phase. Compared with Kitaake plants, dwarf and delayed heading phenotypes were also found in *OsBAG6OE* transgenic plants in reproductive stage (Fig. 6A–C). Yield traits were also impacted in transgenic lines: mature grains produced by overexpression lines had significantly lower grain length, grain width, and hundred-grain-weight than the wild type (Fig. 6D–H). To further investigate the function of OsBAG6 in regulating grain size, yield traits were also assessed in *osbag6* loss-of-function mutants and *OsBAG6* complementation lines. However, grain size and grain weight did not appear to be affected in *osbag6* mutants (Additional file 1: Fig. S4A–E). Taken together, these results indicate that overexpression of *OsBAG6* negatively affects plant height and grain size, impacting grain yield and crop architecture.

### RNA-Seq Analysis of Transcriptional Changes Induced by Overexpression of *OsBAG6*

**Fig. 7:**
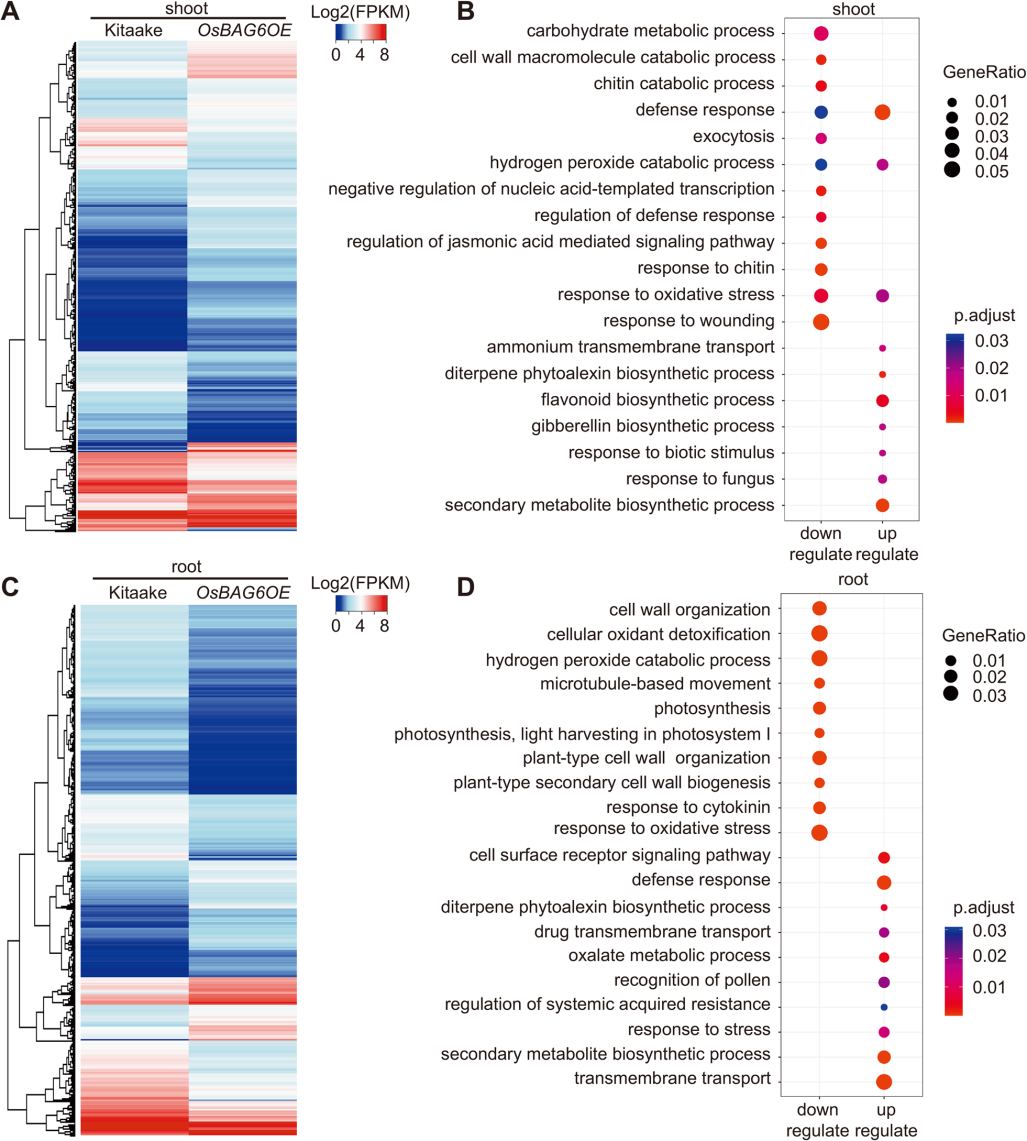
OsBAG6 affects the landscape of transcriptional regulation in rice. **A** Hierarchical clustering analysis of differentially expressed genes (DEGs) in shoot: upregulated genes (URGs) and downregulated genes (DRGs) between wild-type (Kitaake) and *OsBAG6OE*. High expression level displayed as red color, and blue indicates low expression. Color scale is shown base on Log_2_(FPKM). **B** Gene ontology (GO) enrichment analysis of DEGs was performed to categorize function of URGs and DRGs in shoot. **C** Hierarchical clustering analysis of DEGs in root: URGs and DRGs between Kitaake and *OsBAG6OE*. High expression level displayed as red color, and blue indicates low expression. Color scale is shown base on Log_2_(FPKM). **D** GO enrichment analysis of DEGs was performed to categorize function of URGs and DRGs in root

RNA-sequencing (RNA-seq) was used to investigate the impact of *OsBAG6* overexpression of genome-wide transcription. Transcriptomic profiles were compared between Kitaake and transgenic *OsBAG6OE* plants under normal conditions in the shoot and root tissues of 3-week-old plants. Differentially expressed genes (DEGs) were defined as those with adjusted *P*-value (*q*-value) < 0.05 and |log2 fold change|> 1, resulting in the identification of 593 upregulated and 390 downregulated genes (*OsBAG6OE* versus Kitaake) in shoot tissue (Fig. 7A, Additional file 2: Table S2). Gene Ontology (GO) analysis of upregulated genes (URGs) found that the “flavonoid biosynthetic process”, “response to biotic stimulus”, and “hydrogen peroxide catabolic process” categories were enriched in transgenic lines compared to WT. Analysis of downregulated genes (DRGs) showed that the “response to wounding”, “response to oxidative stress”, “hydrogen peroxide catabolic process”, and “carbohydrate metabolic process” categories were relatively enriched (Fig. 7B).

In roots, 1146 upregulated and 2079 downregulated genes were identified (*OsBAG6OE* versus Kitaake) (Fig. 7C, Additional file 2: Table S3). GO analysis of root DEGs showed that the “oxalate metabolic process”, “response to stress”, and “transmembrane transport” categories were relatively enriched in transgenic lines compared to WT, and that the “response to oxidative stress”, “hydrogen peroxide catabolic process”, and “cellular oxidant detoxification” were relatively enriched in WT compared with transgenic lines (Fig. 7D). Among the “transmembrane transport” category in the up-regulated genes in roots, we noticed two genes related to metal transport, *OsYSL2,* and *OsTOM2*. OsYSL2 is a transporter expressed in phloem cells that functions as a carrier for iron (Fe(II)) and manganese (Mn(II)) in the form of nicotianamine complexes (Koike et al. 2004; Ishimaru et al. 2010). OsTOM2 plays a critical role as an efflux transporter for phytosiderophores, which are essential metal chelators utilized by graminaceous (Nozoye et al. 2015). To verify the expression level of these two genes, in addition to *OsBAG6OE*, we also assessed these in *osbag6* mutants. As shown in Additional file 1: Fig. S5A and B, we found the expression level of these two genes was upregulated in the roots of *OsBAG6OE* plants but downregulated in the root of the *osbag6* mutants. We speculate that the expression of these genes influences the transport of Fe, Mn, and Zn in response to saline-alkaline stress.

Most of the DEGs were associated with the GO terms “response to oxidative stress”, “hydrogen peroxide catabolic process”, and “secondary metabolite biosynthetic process” (Fig. 7B, D). Modulation of secondary metabolism is a primary mechanism used by plants to mitigate the impact of environmental stress (Austen et al. 2019). Plant cells also accumulate reactive oxidative species (ROS) as metabolic byproducts, and their accumulation increases during stress metabolic responses (Choudhury et al. 2017; Wang et al. 2020b). Enrichment of genes related to these pathways suggests that *OsBAG6* may participate in plant responses to environmental stress.

### OsBAG6 Interact with OsCaM1-1 in the Absence of Ca^2+^

**Fig. 8:**
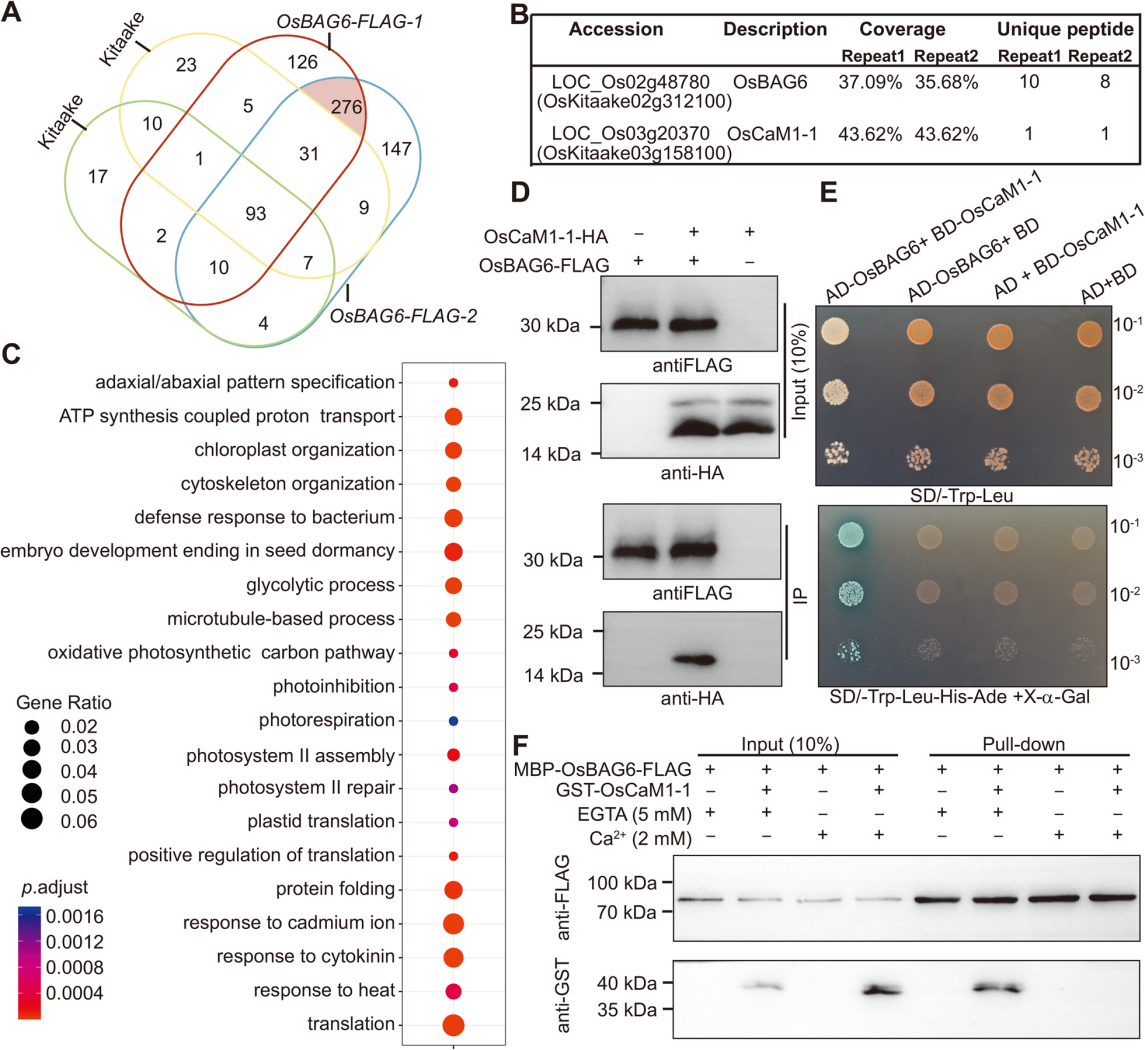
OsBAG6 interacts with OsCaM1-1 both in vivo and in vitro. **A** Venn diagram showing overlap of proteins identified by affinity purification and mass spectrometry analysis in two repeats. **B** OsBAG6 binding proteins identified by IP-MS. The percentage of full-length protein covered by identified unique peptides were defined as Coverage. Unique peptides indicate the number of identified peptides that are mapped to an individual protein. **C** GO enrichment analysis of 276 genes shade in red encoding OsBAG6 and OsBAG6 interacting proteins in Fig. 8A. **D** Immunoblot analysis of the results from the co-immunoprecipitation (co-IP) assay. *OsBAG6-FLAG* construct was co-transfected with *OsCaM1-1-HA* into Kitaake protoplasts. *OsBAG6-FLAG* and *OsCaM1-1-HA* were separately transfected as negative control. Anti-FLAG antibody was used to perform immunoprecipitation, and pulled-down proteins were detected using anti-HA antibody. **E** Yeast two hybrid (Y2H) assay was performed to test the binding between OsBAG6 with OsCaM1-1 in vitro. **F** Pull-down assay was used to test the binding affinity between OsBAG6 with OsCaM1-1 in the presence of 5 mM EGTA or 2 mM Ca^2+^. Magnetic beads attaching anti-FLAG antibody was used for pull-down

OsBAG6 is a chaperone regulator that interacts with a wide range of proteins. To isolate the OsBAG6-interating proteins involved in the saline-alkaline stress response, two independent transgenic lines expressing OsBAG6-FLAG were used for immunoprecipitation-mass spectrometry (IP-MS) analysis. OsBAG6 peptides and 275 OsBAG6-binding proteins were identified from OsBAG6-FLAG expressing plants, including HSP70, which was previously noted as a BAG-interacting protein (Li et al. 2016a; Wang et al. 2020a) (Fig. 8A, B, Additional file 2: Table S4). The 275 interaction partners of OsBAG6 identified by IP-MS were subjected to GO analysis. Enriched categories included “ATP synthesis coupled proton transport” and “glycolytic process”, as well photosynthesis related categories such as “oxidative photosynthetic carbon pathway”, “photoinhibition”, “photorespiration”, “photosystem II assembly”, and “photosystem II repair” (Fig. 8C). These enriched categories suggest that OsBAG6 may have a role in energy biosynthesis and metabolic pathway, which may explain why *OsBAG6OE* plants exhibited decreased plant height and seed size and increased saline-alkaline sensitivity. One of the putative OsBAG6-interating proteins was a Ca^2+^ sensor protein, OsCaM1-1 (Fig. 8B). Calmodulin (CaM) is involved in sensing calcium (Ca^2+^) and participates in Ca^2+^ signaling to modulate sodium transport. OsCaM1-1 proteins were previously shown to have a positive role in salt stress response in rice by mitigating salt-induced oxidative damage (Saeng-ngam et al. 2012; Kaewneramit et al. 2019). OsCaM1-1 is thus a candidate interaction partner for OsBAG6 in the regulation of saline-alkaline stress. The interaction between OsCaM1-1 and OsBAG6 was assessed using co-immunoprecipitation (co-IP). *OsBAG6-FLAG* and *OsCaM1-1-HA* were co-transformed into protoplasts, with solo *OsBAG6-FLAG* or *OsCaM1-1-HA* transfections used as negative controls. As shown in Fig. 8D, OsBAG6 interacts with OsCaM1-1 in vivo. Yeast two-hybrid analysis was used to further evaluate the direct interaction between OsCaM1-1 and OsBAG6. The full-length of OsBAG6 was fused to the activation domain of GAL4 (AD) to produce the *AD-OsBAG6* construct. Full-length OsCaM1-1 was fused to the GAL4 DNA-binding domain. Mating yeast of AD-OsBAG6 and BD-OsCaM1-1 grown on synthetic dropout (SD) medium (SD/-Trp/-Leu/-His/-Ade + X-α-Gal) and turn to blue (Fig. 8E), suggesting that OsBAG6 interacted with OsCaM1-1 in vitro.

In previous studies, Ca^2+^ binding induced conformational changes in calmodulin proteins, and the binding mode changed between calmodulin and IQ domain-containing proteins when Ca^2+^ was applied to the system (Li et al. 2016a, 2017a; Zhang et al. 2017; Wu et al. 2022). It is therefore possible that OsCaM1-1 binding to OsBAG6 might differ in the presence and absence of Ca^2+^. To evaluate this hypothesis, pull-down assays were performed with purified MBP-OsBAG6-FLAG and GST-CaM1-1, with MBP-OsBAG6-FLAG alone as the negative control, in the presence of 5 mM EGTA or 2 mM CaCl_2_. After incubation with magnetic beads attaching anti-FLAG antibody, bound proteins were detected using anti-GST antibody. OsBAG6 interacted with OsCaM1-1 in the absence of Ca^2+^ but did not bind when Ca^2+^ was present (Fig. 8F). In contrast to *Arabidopsis*, where changes in Ca^2+^ only decreased the binding affinity of AtBAG5 and AtCaM (Li et al. 2016a), OsBAG6 was completely unable to interact with Ca^2+^ saturated OsCaM1-1.

Based on these results, we raised a model for the role of OsBAG6 under saline-alkaline stress. Under normal conditions, OsBAG6 interacts with OsCaM1-1. However, under saline-alkaline stress treatment, cytoplastic Ca^2+^ concentration increases and Ca^2+^ saturated OsCaM1-1 cannot interact with OsBAG6. Released OsCaM1-1 then activates downstream genes that respond to the saline-alkaline stress. OsBAG6 also interacted with energy biosynthesis and metabolic pathway proteins that are involved in plant growth and stress response.

## Discussion

### *OsBAG6* is a Novel Negative Regulator of Saline-Alkaline Stress in Rice

BAG proteins in humans are involved in the regulation of a wide range of physiological processes including apoptosis, autophagy, cell cycle and proliferation, immunomodulation, and protein homeostasis (Doong et al. 2002; Behl 2016; Mariotto et al. 2020). The classic functions of BAGs involve association with BCL-2 enhancing the anti-apoptotic effect, and interaction with 70 kDa heat shock protein (HSP70) to function as co-chaperone to control protein homeostasis (Mariotto et al. 2020). As in humans, BAG proteins in plants also participate in multiple biological processes, including plant development pathways and stress responses (Kang et al. 2006; Kabbage et al. 2017; Locascio et al. 2019; Jiang et al. 2023). However, the roles of BAG proteins and their binding partners in the saline-alkaline stress response have not been examined to date. Six BAG proteins were previously identified in rice (You et al. 2016; Zhou et al. 2021). In this study, we propose the smallest of these BAG proteins, OsBAG6, a mitochondrial chaperone regulator, as a novel regulator of saline-alkaline stress in rice. Compared with WT plants, transgenic rice plants constitutively overexpressing *OsBAG6* had significantly lower survival rates under saline-alkaline stress conditions (Fig. 3A, B), whereas *osbag6* loss-of-function mutants exhibited elevated saline-alkaline stress tolerance (Fig. 4D, E). Consistent with the phenotype, transcriptome data indicated that numbers of genes positively regulating saline stress tolerance were downregulated in *OsBAG6* overexpression lines. For example, *OsTPS8* and *OsJAZ9* were downregulated in *OsBAG6OE* plants (Fig. 7, Additional file 2: Table S2). Knock-out of *OsTPS8*, a *TREHALOSE-PHOSPHATE-SYNTHASE* (*TPS*) gene, exhibits a salt sensitive phenotype due to a reduction of soluble sugar, Casparian band and suberin deposition in roots, and downregulation of ABA-responsive genes and SAPKs (rice SnRK2s) (Vishal et al. 2019). Another gene, *OsJAZ9*, a TIFY family gene, interacts with bHLH transcription factors and positively regulate salt stress tolerance by mediating K^+^ homeostasis (Wu et al. 2015). Overall, our results provide evidence that OsBAG6 is a negative regulator of saline-alkaline stress that acts by repressing the expression of positive regulatory proteins. The transcriptome data also showed that *OsDREB1A*, *OsDREB1B*, *OsDREB1C*, *OsDREB1E*, *OsDREB1H,* and *OsDREB1G* were downregulated in shoots of *OsBAG6*-overexpressing plants (Additional file 2: Table S2). Dehydration-responsive element binding proteins (DREBs) are transcription factors that specifically bind with the dehydration-responsive element (G/ACCGAC) to regulate the expression of some stress inducible genes (Dubouzet et al. 2003). OsDREB1A was shown to have a positive role in drought, salt, and cold stress tolerance (Dubouzet et al. 2003), although overexpression of *OsDREB1A* showed growth retardation under the normal condition for 50 days after sowing, while some lines exhibited dwarf phenotype even at the reproductive stage (Ito et al. 2006). The same growth retardation phenotypes were also found in *OsDREB1B* overexpressing lines (Ito et al. 2006). Ectopic expression of *OsDREB1B* in *Arabidopsis* increased cold and heat tolerance (Qin et al. 2007). Overexpression of *OsDREB1C* increased yield and shortened growth duration (Wei et al. 2022). Overexpression of *OsDREB1E* slightly increased drought resistance, and overexpression of *OsDREB1G* dramatically elevated drought tolerance in rice (Chen et al. 2008). Down-regulation of these *OsDREB1s* in *OsBAG6* overexpressing plants may contribute to their saline-alkaline sensitive phenotypes.

Moreover, we also found the expression *OsYSL2* and *OsTOM2* down-regulated in the roots of *osbag6* mutant and up-regulated in *OsBAG6OE* plants. OsYSL2 plays a role in iron and manganese transport. Knockdown of *OsYSL2* results in the decrease of Fe and Mn concentration in shoots, and the increase of Fe in roots during the vegetative stage. In iron-deficient plants, the expression of *OsYSL2* was induced in the leaves (Koike et al. 2004; Ishimaru et al. 2010). OsTOM2 plays a critical role as an efflux transporter for phytosiderophores, which are essential metal chelators utilized by graminaceous. Knockdown of *OsTOM2* results in the Fe, Mn, Zn, and Cu concentrations increased in the leaves or roots of rice. The expression level of *OsTOM2* was induced by Fe deficiency (Nozoye et al. 2015). The differential expressed genes affected by OsBAG6, which functions in metal ion transport may also contribute to the saline-alkaline stress response.

### Potential Functions of OsBAG6 in Saline-Alkaline Stress Tolerance

BAG proteins are chaperone regulators that coordinate with chaperone or other proteins to perform diverse functions, including plant development and stress response functions (Kabbage et al. 2016; Wang et al. 2020a). AtBAG1 together with Hsc70 to mediate proteasomal degradation of unimported plastid proteins, plays an negative role in salt stress response, supporting by the growth of *AtBAG1*-overexpressing plants had more retarded phenotype from the early stage of growth compared with the empty vector expression lines in high salt conditions (Lee et al. 2016). OsBAG4 interacts with OsMYB106 (a transcription factor) and OsSUVH7 (a DNA methylation reader). OsMYB106 binds to the promoter region of *OsHKT1;5* and recruits OsSUVH7 to the methylated MITE region (transposon) in the promoter region of *OsHKT1;5*. OsBAG4 stabilizes this complex to positively respond to saline stress (Wang et al. 2020a). In this study, OsCaM1-1 was identified as a novel co-factor interacting with OsBAG6 (Fig. 8, Additional file 2: Table S4). OsCaM1-1 enhanced the lateral root growth via auxin content in roots under saline stress (Yang et al. 2021). Under normal condition, the expression level of auxin-related genes (*OsPIN9, OsAUX1*, and *OsIAA11*) were higher in *OsCaM1-1* overexpression line and lower in *OsCaM1-1* antisense-RNA repression expression lines, which demonstrating that OsCaM1 positively regulated lateral root growth through auxin signaling under stress in early rice seedlings (Yang et al. 2021). In our transcriptome data, *OsAUX4*, *OsAUX5*, *OsIAA2*, *OsIAA7*, *OsIAA11*, *OsIAA12*, *OsIAA13*, *OsIAA24*, *OsIAA30*, *OsIAA31*, *OsPIN1*, and *OsPIN2* were downregulated in roots of *OsBAG6OE* (Additional file 2: Table S3). *OsBAG6OE* plants exhibited saline-alkaline sensitive phenotype as *OsCaM1-1* antisense-RNA repression expression lines, which displayed sensitive phenotype under saline stress. Previous study has reported that OsCaM1-1 extensively influenced the transcript level of genes participated in many cellular processes, including transcription, hormone-mediated regulation, signaling, secondary metabolism, photosynthesis and so on (Yuenyong et al. 2018). Under normal conditions, 80% of the DEG between *OsCaM1-1* overexpression line with WT were salt stress-responsive, which mean a possible effect on the saline stress response of the overexpression of *OsCaM1-1* (Yuenyong et al. 2018). These results suggest that OsBAG6 interacts with OsCaM1-1 to regulate the expression of genes involved in saline stress response and genes involved in auxin signaling, responding to saline-alkaline stress tolerance.

### Possible Functions of OsBAG6 in Regulation of Seed Size, Seedling Height, and Heading Date

Seed size and seeding height are key agronomic traits that affect rice yield. In transgenic *OsBAG6-*overexpressing plants, significant decreases were found in seed size and seedling height, and flowering was delayed (Fig. 6). IP-MS analysis identified several photoperiod pathway proteins, including GLO1, GLO4, and OsPhyB, which interacted with OsBAG6 (Additional file 2: Table S4). GLO1 and GLO4 are glycolate oxidase proteins that act as important enzymes during photorespiration in rice. GLO1 and GLO4 were also found to provide an alternative source for H_2_O_2_ production in rice (Zhang et al. 2016). OsPhyB is one of the three phytochromes in rice: phytochromes are the only photoreceptors for perceiving red/far light in rice (Takano et al. 2005, 2009). *OsPhyB* expression reduced the transcription level of floral repressor *Ghd7*, thereby mediating the photoperiodic control of flowering in rice (Osugi et al. 2011). The binding partners of OsBAG6 are also clustered in ATP synthesis coupled proton transport (Fig. 8C), which include ATP synthase B chain, C chain, r subunit, gamma chain, F_1_ (delta subunit family proteins) (Additional file 2: Table S4). ATP synthase structure in mammals has been studied widely. ATP is produced from ADP through ATP synthases, which are present in the inner membrane and cristae of mitochondria, the thylakoid membrane of chloroplasts, and the plasma membrane of bacteria (Jonckheere et al. 2012). While it is commonly known that ATP generation occurs in mitochondria, human mitochondrial ATP synthase (or Complex V) is made up of two functional domains: F_1_ and F_o_. F_o_ facilitates the transfer of protons from the intermembrane space to the matrix, where the energy is created by the proton electrochemical gradient. F_1_ then phosphorylates ADP to produce ATP (Jonckheere et al. 2012). BAG6 interacts with the subunit or chain of ATP synthase, which may affect the proton transport. A mitochondrial ATP synthase small subunit gene (*RMtATP6*) has been reported in rice, overexpression of *RMtATP6* results in greater tolerance to salt stress at the seedling stage in tobacco (Zhang et al. 2006). We speculated that the overexpression of *OsBAG6* occupied more ATP synthase, so the proton transport was affected, and ATP synthesis was hindered, which resulted in lower plant height and saline-alkaline stress-sensitive phenotype. These binding partners may indicate a role for OsBAG6 in photoperiod perception and regulation, which would impact seedling height, heading date and seed size as well as flowering time.

### Potential Application of OsBAG6 in Rice Adaptation Improvement

Poor irrigation and cultural practices, low precipitation, climate change, and native rock weathering are contributing to an increase in the global area of saline-alkaline soils at an annual rate of 10% (Shrivastava and Kumar 2015). Soil saline-alkalization is an widespread abiotic stressor that has turned into a primary limiting factor in global agriculture for crop production (Wang et al. 2018). Breeding varieties with high resistance to saline-alkaline stress, preferably without compromising agronomic traits, is an important strategy for management of challenging soil conditions. OsBAG6 is a negative regulator of saline-alkaline stress tolerance and is therefore a strong potential target gene for rice adaptation improvement and resistance breeding. Saline-alkaline stress was enhanced in *osbag6* mutants with no significant decrease in grain size and grain weight. Further dissection of *OsBAG6* functions will facilitate the development of high saline-alkaline stress resistant rice cultivars without loss of yield.

## Conclusions

In this study, we demonstrated a mitochondrial localized chaperone OsBAG6 (OsKitaake02g312100) in rice was involved in saline-alkaline stress tolerance. Overexpression of *OsBAG6* decreased saline-alkaline stress tolerance, as well as plant height, grain size. *osbag6* mutant exhibited saline-alkaline stress tolerance phenotype with no significant alteration in grain size. Revealing that OsBAG6 and OsCaM1-1 dissociated in the presence of calcium ions, and subsequently impact the expression level of downstream genes, which involved in stress response. OsBAG6 also interact with some energy biosynthesis and metabolic proteins to regulate plant growth and stress response. This study broadens the gene function of the rice BAG family, especially in subfamily II, and reveals that OsBAG6 and OsCAM1-1 act together to participate in the regulation of saline-alkaline stress tolerance.

### Supplementary Information

**Additional file 1**: **Fig. S1** Construction of *OsBAG6* overexpression lines. **Fig. S2** Characterization of *OsBAG6* overexpression lines and *osbag6* mutants under saline-alkaline stress in soil-based conditions. **Fig. S3** Characterization of *OsBAG6* overexpression lines and *osbag6* mutants under salt stress conditions. **Fig. S4** Grain traits of *osbag6* mutants. **Fig. S5** Expression level of *OsYSL2* and *OsTOM2*.

**Additional file 2**: **Table S1** Primers used in this study. **Table S2** List of differentially expressed genes (DEGs) in shoots in *OsBAG6OE* vs. Kitaake under normal conditions. **Table S3** List of differentially expressed genes (DEGs) in roots in *OsBAG6OE* vs. Kitaake under normal conditions. **Table S4** Specifically enriched proteins in OsBAG6-FLAG expressed plants.

## Data Availability

RNA-seq data generated in this study have been deposited in the National Center for Biotechnology Information (NCBI) Sequence Read Archive (SRA) database under the accession number PRJNA977860. All data generated or analysed during this study are included in this published article and its supplementary information files.

## Abbreviations

BAG: B-cell lymphoma 2 (Bcl-2)-associated athanogene
CaM: Calmodulin
IQ motif: Calmodulin-binding domain/isoleucine glutamine
ABA: Abscisic acid
CRISPR: Clustered Regularly Interspaced Short Palindromic Repeats
CDS: Coding sequence
AD: Activation domain of GAL4
BD: DNA-binding domain of GAL4
RT-qPCR: Real-time quantitative PCR
DEGs: Differentially expressed genes
GO: Gene Ontology
URGs: Upregulated genes
DRGs: Downregulated genes
ROS: Reactive oxidative species
DAB: Diaminobenzidine
WT: Wild-type
