# Supplementary material for: A Mitochondrial Localized Chaperone Regulator OsBAG6 Functions in Saline-Alkaline Stress Tolerance in Rice
Source: Rice (N Y). 2024 Jan 22;17:10. doi: 10.1186/s12284-024-00686-z (PMC10803725; doi:10.1186/s12284-024-00686-z)
Supplement: Supplementary file 1 — Additional file 1: Fig. S1 Construction of OsBAG6 overexpression lines. Fig. S2 Characterization of OsBAG6 overexpression lines and osbag6 mutants under saline-alkaline stress in soil-based conditions. Fig. S3 Characterization of OsBAG6 overexpression lines and osbag6 mutants under salt stress conditions. Fig. S4 Grain traits of osbag6 mutants. Fig. S5 Expression level of OsYSL2 and OsTOM2. [file 12284_2024_686_MOESM1_ESM.docx]

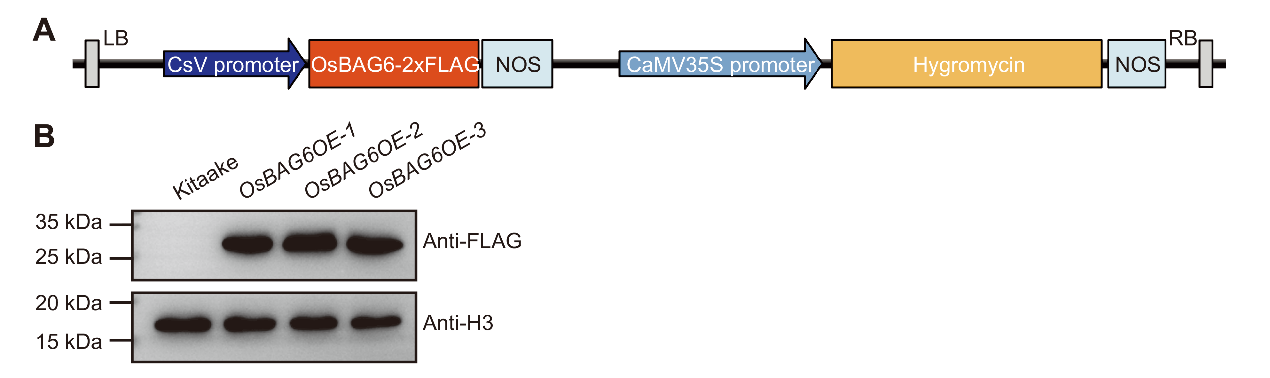


**Fig. S1** **Construction of *OsBAG6* overexpression lines.** (A) Diagrams of the overexpression plasmid of *OsBAG6*. (B) Immunoblot analysis of OsBAG6-FLAG in Kitaake, *OsBAG6OE-1*, *OsBAG6OE-2,* and *OsBAG6OE-3* using anti-FLAG antibody. Histone H3 was used as a loading control.


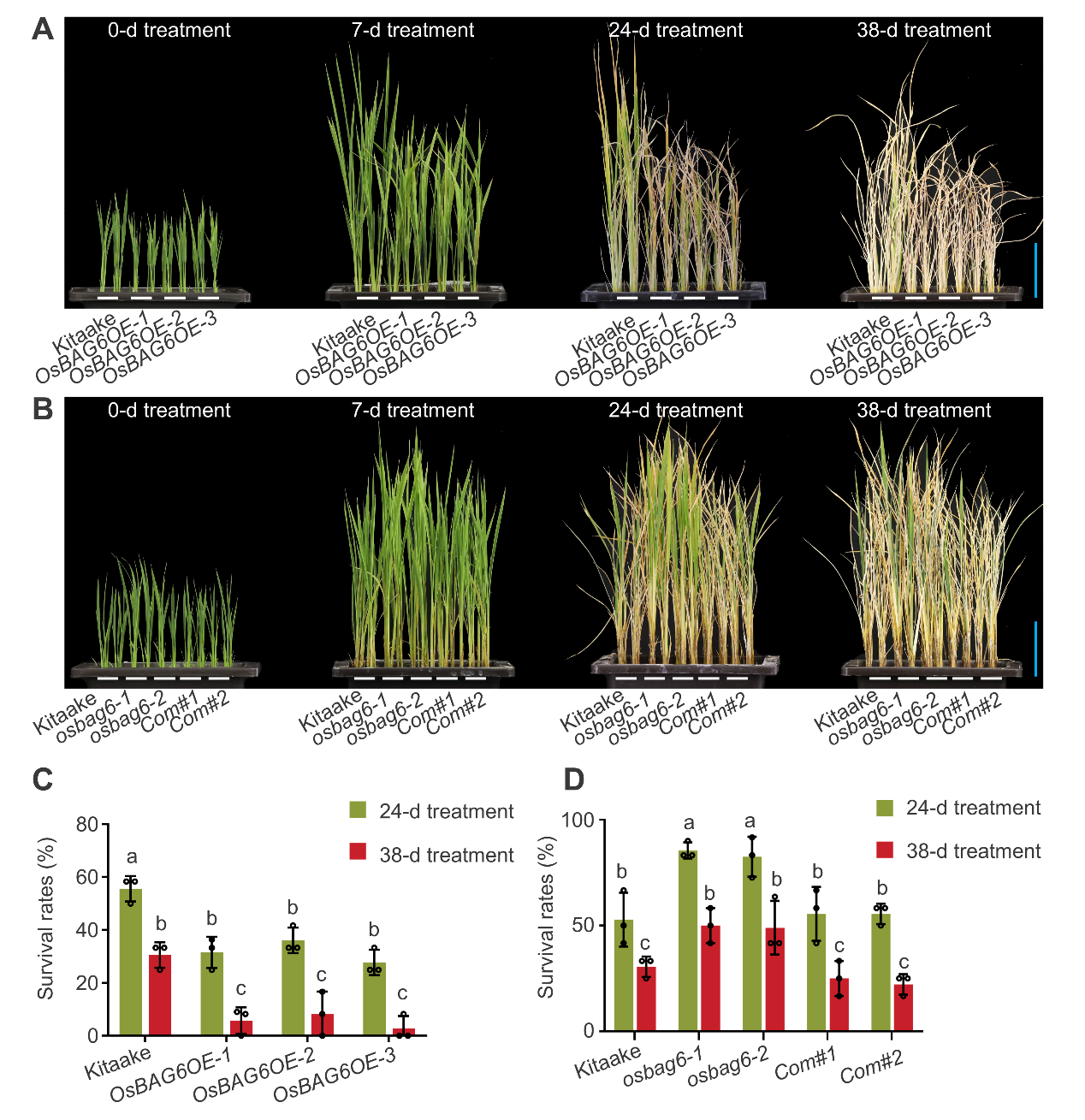


**Fig. S2 Characterization of *OsBAG6* overexpression lines and *osbag6* mutants under saline-alkaline stress in soil-based conditions.** (A, B) Image of Kitaake and *OsBAG6OE* lines (A) and Kitaake, *osbag6* mutants and *OsBAG6* complementation lines (*Com#1* and *Com#2*) (B) before and after 7 d, 24 d, and 38 d treatment of 25 mM Na_2_CO_3_ (dissolved in water, pH ≈ 11.2, Na^+^ = 50 mM). Scale bar (blue) = 5 cm. (C, D) Survival rates of Kitaake and *OsBAG6OE* plants (C) and Kitaake, *osbag6* mutants and *OsBAG6* complementation lines (D) after 24-d and 38-d treatment of saline-alkaline stress. Data represent mean ± SD of 3 replicates (*n* = 3, 12 plants per genotype were used to calculate survival rate per replicate). Significant differences were evaluated by two-way ANOVA, followed by Tukey’s multiple comparison test.

**Fig. S3 Characterization of *OsBAG6* overexpression lines and *osbag6* mutants under salt stress conditions.** (A, B) Image (A) and survival rates (B) of Kitaake and 3 independent *OsBAG6OE* lines before and after 3-d recovery from salt stress condition (100 mM NaCl). In (A), scale bar = 8 cm. In (B), Data represent mean ± SD of 5 replicates (*n* = 5, 32 plants per genotype were used to calculate survival rate per replicate). Significant differences were evaluated by two-way ANOVA, followed by Tukey’s multiple comparison test. (C, D) Image (C) and survival rates (D) of Kitaake and 2 independent *osbag6* mutants before and after 3-d recovery from salt stress condition (100 mM NaCl). In (C), scale bar = 8 cm. In (D), Data represent mean ± SD of 5 replicates (*n* = 5, 32 plants per genotype were used to calculate survival rate per replicate). Significant differences were evaluated by two-way ANOVA, followed by Tukey’s multiple comparison test.

**
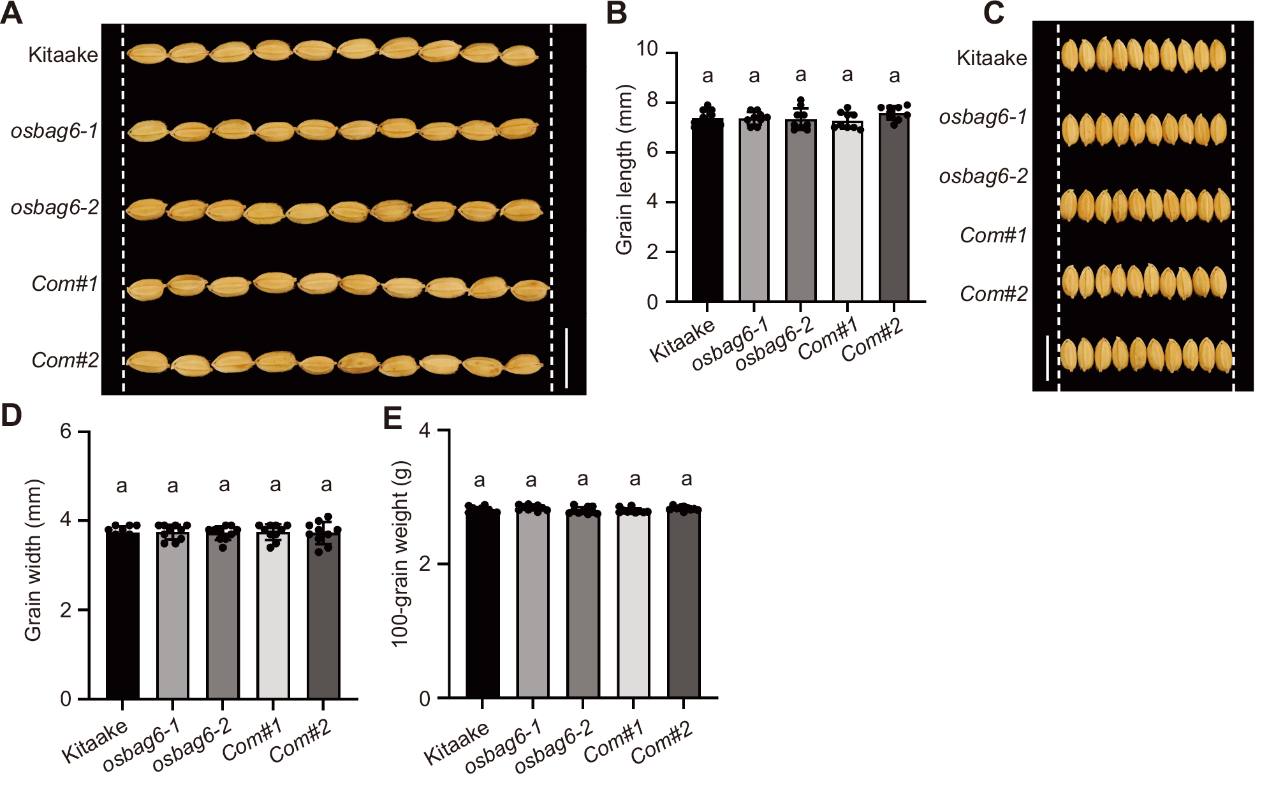
**

**Fig. S4** **Grain traits of *osbag6* mutants.** (A, B) Image (A) and measurements (B) of grain length in the indicated genotypes. Scale bar = 1 cm, Data represent mean ± SD of 10 biological replicates. (C, D) Image (C) and measurements (D) of grain width in the indicated genotypes. Scale bar = 1 cm, Data represent mean ± SD of 10 biological replicates. (E) Measurements of 100-grain weight in the indicated genotypes. Data represent mean ± SD of 10 biological replicates (each contains 100 grains). Significant differences were evaluated by one-way ANOVA, followed by Tukey’s multiple comparison test.

**
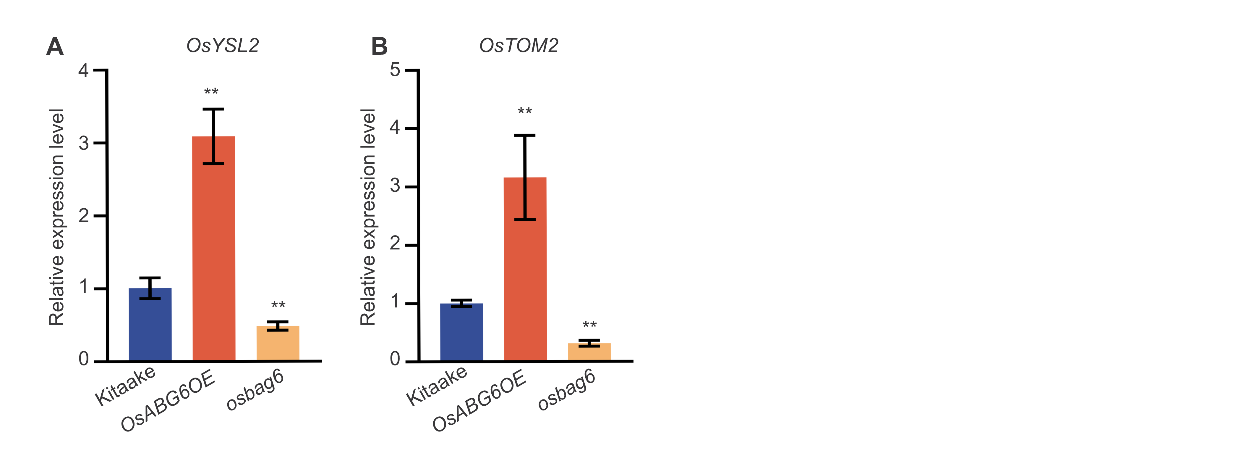
**

**Fig. S5 Expression level of *OsYSL2* and *OsTOM2*.** (A) The relative expression level of *OsYSL2* in the roots of Kitaake, *OsBAG6OE,* and *osbag6* mutants, as determined by real-time quantitative PCR (RT-qPCR). (B) The relative expression level of *OsTOM2* in the roots of Kitaake, *OsBAG6OE,* and *osbag6* mutants. *OsGAPDH1* was used as the internal control. Error bars indicate ± SD (*n* = 3). ** represent *p* < 0.05.
